# Service-Use Subtypes and Caregiver Burden in Home-Living Adults with Severe Care Needs in Japan

**DOI:** 10.1093/geroni/igaf122.4007

**Published:** 2025-12-31

**Authors:** Yu Sun, Masao Iwagami, Nobuo Sakata, Satoru Yoshie, Yoko Hamasaki, Shoichi Masumoto, Gen Nakayama, Ryohei Goto

**Affiliations:** University of Tsukuba, Tsukuba, Ibaraki, Ibaraki, Japan; University of Tsukuba, Tsukuba, Ibaraki, Ibaraki, Japan; University of Tsukuba, Tsukuba, Ibaraki, Ibaraki, Japan; University of Tsukuba, Tsukuba, Ibaraki, Ibaraki, Japan; University of Tsukuba, Tsukuba, Ibaraki, Ibaraki, Japan; University of Tsukuba, Tsukuba, Ibaraki, Ibaraki, Japan; University of Tsukuba, Tsukuba, Ibaraki, Ibaraki, Japan; University of Tsukuba, Tsukuba, Ibaraki, Ibaraki, Japan

## Abstract

**Objectives:**

As Japan transitions from hospital- to community-centered care, the burden on family caregivers is a growing concern. We aimed to identify subgroups of community-dwelling adults with severe care needs based on patterns of medical and long-term care (LTC) service use and to examine associations with caregiver burden.

**Methods:**

We conducted a cross-sectional survey in four municipalities of Ibaraki Prefecture in Japan, targeting dyads of care managers and primary caregivers of adults with care-need level ≥3 living at home. Latent class analysis (LCA) was applied to service-use indicators from care manager questionnaires. Then, caregiver questionnaires were linked to assess high caregiver burden, defined by the Burden Index of Caregivers-11 (BIC-11) and dichotomized at the median. Additional analyses examined five BIC-11 domains (time-dependent, emotional, existential, physical, service-related). Adjusted prevalence ratios (aPRs) were estimated using modified Poisson regression with robust variance, adjusting for patient and caregiver covariates.

**Results:**

Of 320 care manager respondents, LCA identified three subtypes: (1) outpatient/day services–centered, (2) multi-component home-visit services (mainly physician and nurse home visits), and (3) outpatient/day rehabilitation–centered. After linkage and exclusion of missing data, 243 dyads were analyzed. Compared with the outpatient/day services subtype, the multi-component home-visit subtype was associated with a lower prevalence of high caregiver burden (aPR 0.66; 95% CI 0.46–0.95). Domain-specific analyses showed significantly lower emotional and existential burden in this group.

**Conclusions:**

Among adults with severe care needs at home, a multi-component home-visit service pattern was associated with lower caregiver burden, suggesting that appropriate service combinations may help mitigate caregiver strain.

